# Different pattern of CSF glial markers between dementia with Lewy bodies and Alzheimer’s disease

**DOI:** 10.1038/s41598-019-44173-8

**Published:** 2019-05-24

**Authors:** Estrella Morenas-Rodríguez, Daniel Alcolea, Marc Suárez-Calvet, Laia Muñoz-Llahuna, Eduard Vilaplana, Isabel Sala, Andrea Subirana, Marta Querol-Vilaseca, María Carmona-Iragui, Ignacio Illán-Gala, Roser Ribosa-Nogué, Rafael Blesa, Christian Haass, Juan Fortea, Alberto Lleó

**Affiliations:** 1 Memory Unit, Neurology Department, Hospital de la Santa Creu i Sant Pau, Barcelona, Spain; 2 Institut d’Investigacions Biomediques Sant Pau – Universitat Autònoma de Barcelona, Barcelona, Spain; 3 Centro de Investigación Biomédica en Red en Enfermedades Neurodegenerativas, CIBERNED, Instituto de Salud Carlos III, Barcelona, Spain; 4 Chair of Metabolic Biochemistry, Biomedical Center (BMC), Faculty of Medicine, Ludwig-Maximilians-Universität München, Munich, Germany; 5 German Center for Neurodegenerative Diseases (DZNE) Munich, Munich, Germany; 6 Munich Cluster for Systems Neurology (SyNergy), Munich, Germany

**Keywords:** Biomarkers, Cognitive ageing, Dementia

## Abstract

The role of innate immunity in dementia with Lewy bodies (DLB) has been little studied. We investigated the levels in cerebrospinal fluid (CSF) of glial proteins YKL-40, soluble TREM2 (sTREM2) and progranulin in DLB and their relationship with Alzheimer’s disease (AD) biomarkers. We included patients with DLB (n = 37), prodromal DLB (prodDLB, n = 23), AD dementia (n = 50), prodromal AD (prodAD, n = 53), and cognitively normal subjects (CN, n = 44). We measured levels of YKL-40, sTREM2, progranulin, Aβ_1–42_, total tau (t-tau) and phosphorylated tau (p-tau) in CSF. We stratified the group DLB according to the ratio t-tau/Aβ_1–42_ (≥0.52, indicative of AD pathology) and the A/T classification. YKL-40, sTREM2 and progranulin levels did not differ between DLB groups and CN. YKL-40 levels were higher in AD and prodAD compared to CN and to DLB and prodDLB. Patients with DLB with a CSF profile suggestive of AD copathology had higher levels of YKL-40, but not sTREM2 or PGRN, than those without. T+ DLB patients had also higher YKL-40 levels than T−. Of these glial markers, only YKL-40 correlated with t-tau and p-tau in DLB and in prodDLB. In contrast, in prodAD, sTREM2 and PGRN also correlated with t-tau and p-tau. In conclusion, sTREM2 and PGRN are not increased in the CSF of DLB patients. YKL-40 is only increased in DLB patients with an AD biomarker profile, suggesting that the increase is driven by AD-related neurodegeneration. These data suggest a differential glial activation between DLB and AD.

## Introduction

Epidemiological, pathological and genetic studies support the importance of the innate immunity in the pathophysiology of neurodegenerative diseases such as Alzheimer’s disease (AD) and Parkinson’s disease (PD)^1,2^. In particular, astroglia and microglia play an important role in neurodegeneration^3,4^. These two cellular types have very different functions in the central nervous system (CNS): microglia, the resident monocytic cells in the CNS, phagocyte cellular debris and protein aggregates, while astrocytes support neuronal and synaptic activities among other key functions^3,4^.

YKL-40 protein, also known as chitinase 3-like 1 protein, is a glycoprotein expressed by astrocytes near amyloid plaques in AD human brain^5,6^. YKL-40 can be detected in cerebrospinal fluid (CSF) and the levels are increased in preclinical and prodromal AD, as well as in other neurodegenerative conditions, such as Frontotemporal Lobar Degeneration (FTLD), Amyotrophic Lateral Sclerosis (ALS) or Multiple Sclerosis (MS)^7–10^.

Other studies have implicated the triggering receptor expressed on myeloid cells 2 (TREM2) receptor in neurodegenerative diseases^11^. Rare heterozygous variants in *TREM2* have been linked with an increased risk of AD^12,13^. Furthermore, recent studies have shown an elevation in the CSF of the soluble fragment of TREM2 (sTREM2) in early stages of sporadic AD^14–16^ as well as in autosomal dominant AD^17^.

Another line of evidence that supports the role of inflammation in neurodegenerative conditions implicates the Progranulin protein (PGRN). PGRN is expressed in many tissues and cell types^18^. In CNS, PGRN is mainly expressed in neurons and microglia^18,19^ where is involved in the mechanisms of cell proliferation and neuroinflammation. PGRN levels are decreased in CSF and blood of patients with heterozygous mutations in the granulin gene (*GRN)*, that are associated with FTLD with TAR-DNA-binding protein 43 inclusions^20–23^. Furthermore, genetic variants that modulate *GRN* expression, such as the *GRN*rs5848 polymorphism, have been associated with an increased risk of AD^24,25^.

There are multiple evidences that support that glia is activated in synucleinopathies^26^. In particular, activated microglia targeting dopamine nigral neurons has been described in PD^27^. Microglial activation in PD and dementia with Lewy bodies (DLB) has been implicated in the initiation and progression of the disease by means of secretion of pro-inflammatory cytokines and reactive oxygen species^26^. In addition, synuclein released from neurons in PD and DLB can be endocytosed by astrocytes forming glial inclusions^27–29^. These inclusions can induce changes in gene expression in astroglia, enhancing the inflammatory response and promoting neurodegeneration^27,29^.

In this study, we investigated the CSF profile of YKL-40, sTREM2, PGRN in patients with DLB and prodromal DLB, and compared this pattern with that of AD. We also examined the influence of concomitant AD pathology on these biomarkers in DLB.

## Results

### Demographics and core CSF biomarkers

Table 1 summarizes the demographics and CSF biomarker values of all the study participants. There were no significant differences between groups in gender, but CN subjects were significantly younger than the other groups. As expected, MMSE scores were lower in DLB and AD than in CN or groups with prodAD and prodDLB. Core AD CSF biomarkers also differed between groups (Table 1). DLB groups had lower levels of Aβ_1–42_ than CN but higher than AD groups. DLB groups had also higher levels of t-tau and p-tau than CN, but lower than AD groups. Frequency of *APOE*ε4 allele in DLB groups was similar to CN and lower than AD groups (p = 0.001).

**Table 1:** Demographic and CSF biomarker data.

		CN (n = 44)	DLB (n = 37)	Prodromal DLB (n = 23)	AD (n = 50)	Prodromal AD (n = 53)	Total	p-value
	Age, y ± SD (range)^a^	67.4 ± 5.1 (60.2–78.7)	76.5 ± 5 (64–84.6)	76.5 ± 6.4 (58.5–85.8)	74.6 ± 5.6 (62.4–86.8)	72.3 ± 6.3 (60.4–85)	73 ± 6.5 (58.5–86–8)	<0.001
	Sex, Female % (n)	56.8 (25)	54.1 (20)	56.5 (13)	62 (31)	60.4 (32)	57.8% (78)	0.949
	*APOE*ε4, %^*^ (n)^b^	18.2 (8)	24.3 (9)	34.8 (8)	58 (29)	75 (39)	45.1 (93)	0.003
	MMSE ± SD^c^	28.9 ± 1.2	23 ± 4.6	26.1 ± 2.4	22.5 ± 3.4	26.7 ± 2.3	25.7 ± 3.8	<0.001
Core AD biomarkers	CSF Aβ_1–42_, pg/mL ± SD^d^	918.2 ± 212.2	602.7 ± 269.2	634 ± 197.7	384.7 ± 105.6	458.1 ± 72.2	583.6 ± 261	<0.001
	CSF t-tau, pg/mL ± SD^e^	228.8 ± 52.3	448.9 ± 333.9	371.3 ± 174.5	694.5 ± 321	609 ± 267.7	493.8 ± 310	<0.001
	CSF p-tau, pg/mL ± SD^f^	45.5 ± 10.2	68.8 ± 42.3	62.6 ± 24.4	94.1 ± 26.2	94.8 ± 39.2	76 ± 36.8	<0.001
Inflammation-related biomarkers	CSF YKL-40, ng/mL ± SD^g^	238.8 ± 49.2	278.8 ± 83.4	270.7 ± 69	295.3 ± 54.1	296.7 ± 55.7	277.8 ± 64.8	<0.001
	CSF sTREM2, ng/mL ± SD (n)^h^	4.2 ± 2.3 (40)	5.3 ± 2.3 (28)	4.4 ± 1.9 (18)	4.3 ± 2.2 (36)	5 ± 2.4 (41)	4.6 ± 2.3 (163)	0.038
	CSF PGRN, ng/mL ± SD	4.3 ± 1.2	4.2 ± 1.1	4.5 ± 1.3	4.4 ± 1.3	4.6 ± 1.2	4.4 ± 1.2	0.653

^*^At least one *APOE*ε4 allele.

^a^Cognitively normal controls (CN) vs. DLB, prodDLB, AD and prodAD, p < 0.001; prodAD vs. prodDLB and DLB, p = 0.05.

^b^CN, DLB and pDLB vs. AD and pAD, p < 0.001.

^c^CN vs. AD, p < 0.001; prodDLB vs. AD, p = 0.09.

^d^CN vs. DLB, prodDLB, AD and prodAD and AD vs. DLB and prodDLB, p < 0.001; AD vs. prodAD, p = 0.003.

^e^CN vs. DLB, AD and prodAD, p < 0.001; CN vs. prod DLB, p = 0.006; DLB vs. AD and prodAD, p < 0.001; prod DLB vs. AD and prod AD, p < 0.001.

^f^CN vs. AD and prodAD, p < 0.001; CN vs. DLB, p = 0.016, CN vs. prodDLB, p = 0.06; DLB and prodDLB vs. AD and prodAD, p < 0.001.

^g^CN vs. AD and prodAD, p < 0.01; DLB and prodDLB vs. AD, p = 0.03; DLB and prodDLB vs. prodAD, p = 0.006 and p = 0.007, respectively.

^h^pAD vs. AD, p = 0.06 (results adjusted by multiple comparisons).

Analyses using ANCOVA including age for all biomarkers and also sex in the case of sTREM2. In post-hoc analyses p-values were adjusted by Bonferroni correction for multiple comparisons (10 comparisons for this analysis).

### Relationship between glial biomarkers, age, *APOE* and clinical measures

There was no association between gender and any of the three glial markers, but there was a trend towards higher levels of sTREM2 in males (p = 0.06). Therefore, all sTREM2 analyses were adjusted by gender. Age significantly correlated with CSF levels of YKL-40 and sTREM2 (r = +0.351; p < 0.001 and +0.212; p < 0.006, respectively) in the whole sample as previously reported^14,17^, without differences when stratifying by diagnosis. We did not find differences in the levels of any of the glial markers between *APOE*ε4 carriers and non-carriers in the whole group. However, in prodDLB, non-carriers showed higher levels of sTREM2 than carriers (5.14 ± 1.9 vs. 3.1 ± 1.1 ng/mL, p = 0.02). sTREM2 levels were not influenced by *APOE*ε4 status in any other clinical group. We did not detect differences in YKL-40 or PGRN levels between *APOE*ε4 carriers and non-carriers in any clinical group.

We did not find any significant association between the levels of glial markers and the clinical or neuropsychological measures after correction for multiple comparisons.

### Glial biomarkers across clinical diagnoses

**Figure 1:**
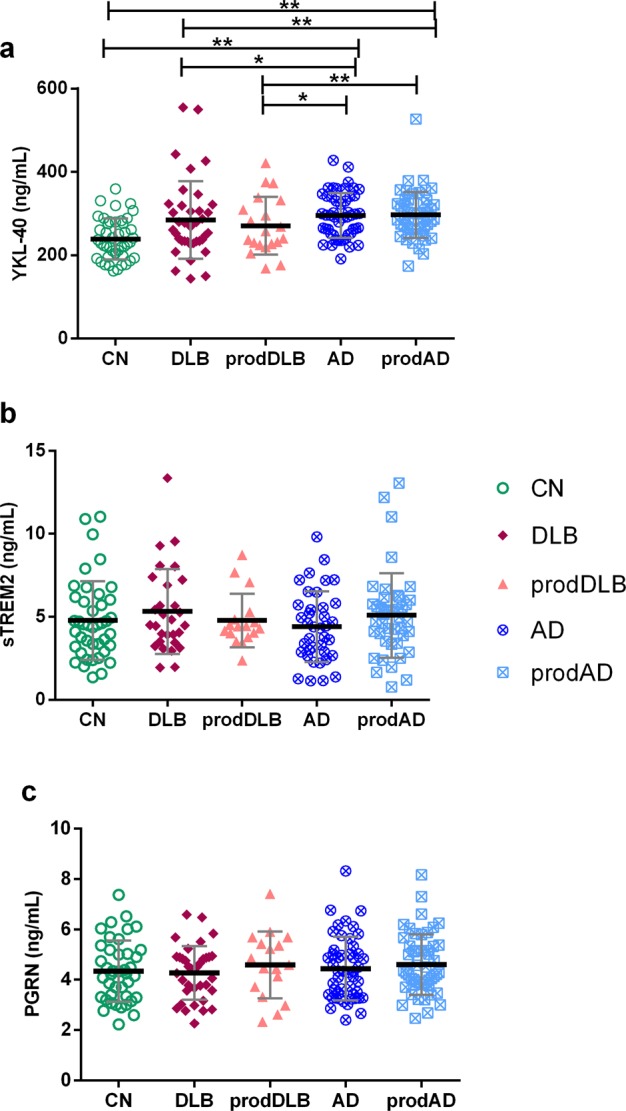
Inflammation-related biomarkers across clinical diagnoses. *p < 0,05, **p < 0.01. The group-wise comparisons were analyzed by ANCOVA adjusting by age for all biomarkers and additionally for sex in the case of sTREM2. The p-values were adjusted by Bonferroni correction for multiple comparisons (10 comparisons). Thicker horizontal bars represent the mean while whiskers represent the standard deviation. CN: cognitively normal controls, DLB: Dementia with Lewy Bodies, prodDLB: prodromal DLB, AD: Alzheimer’s disease, prodAD: prodromal AD. (**a**) CSF YKL-40 levels in the different clinical groups. (**b**) CSF sTREM2 levels in the different clinical groups. (**c**) CSF PGRN levels in the different clinical groups.

Next, we analyzed differences in levels of YKL-40, sTREM2 and PGRN across groups (Fig. 1, Table 1). We did not find significant differences between DLB, prodDLB and CN in the levels of YKL-40 in CSF. Patients with DLB and prodDLB had lower YKL-40 levels than those with AD (both p = 0.03) and prodAD patients (p = 0.006 and p = 0.007, respectively). AD and prodAD had significant increased levels of YKL-40 compared with CN (both, p < 0.01).

sTREM2 levels did not differ between DLB groups and CN, but levels were higher in both, DLB and prodAD, compared to AD (p = 0.02 and p = 0.006, respectively). These differences, however, disappeared when adjusting by Bonferroni correction for multiple comparisons and only a trend towards higher sTREM2 levels in prodAD compared to AD (p = 0.06) was observed. No differences were found across groups in CSF PGRN levels.

### Correlations between glial biomarkers

**Figure 2:**
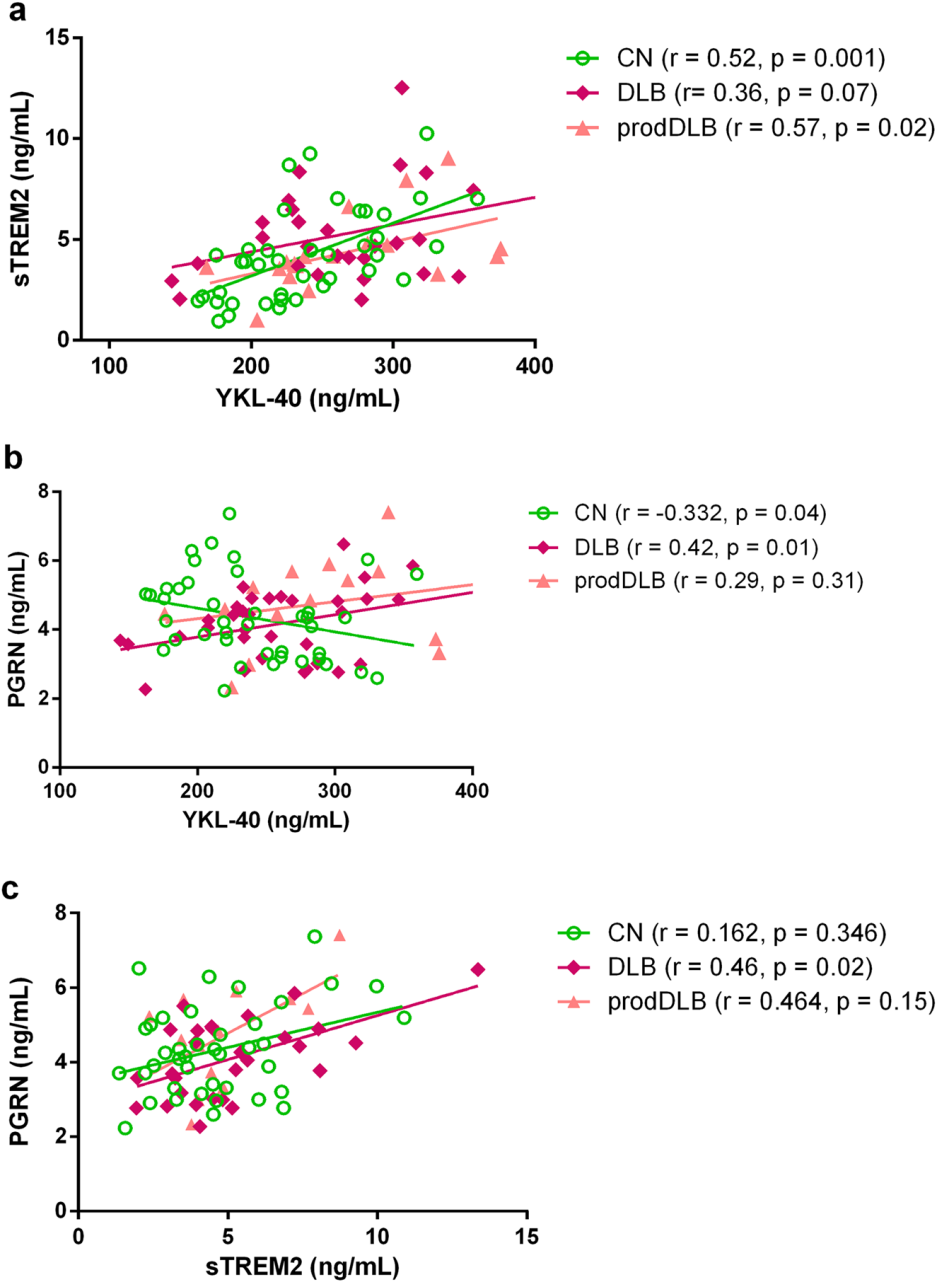
Correlations between glial biomarkers. CN: cognitively normal controls, DLB: Dementia with Lewy Bodies, prodDLB: prodromal DLB, AD: Alzheimer’s disease, prodAD: prodromal AD. (**a**–**c**) Correlations between CSF sTREM2 and YKL-40, PGRN and YKL-40, and sTREM2 and PGRN levels in the different diagnostic groups, r and p-values of the partial correlation are shown in brackets (adjusted by age for all the biomarkers and additionally for sex in sTREM2).

Figure 2 and Table 2 show the correlations between the three investigated glial markers. Supplementary Fig. S1 shows the correlations stratified by diagnosis. We found a correlation between YKL-40 and PGRN levels (r = 0.42, p = 0.01, Fig. 2b) and between sTREM2 and PGRN (r = 0.46, p = 0.02, Fig. 2c) in DLB. We also found that YKL-40 correlated with sTREM2 (r = 0.57, p = 0.02, Fig. 2a) in prodDLB. In AD groups, we found correlations between YKL-40 and PGRN and sTREM2 and PGRN only in prodAD (Fig. 2b,c), but not in AD patients, while YKL-40 and sTREM2 were correlated in both AD and prodAD (Fig. 2a).

**Table 2:** Partial correlations between glial markers across diagnoses.

	CN (n = 44)	DLB (n = 37)	Prodromal DLB (n = 23)	AD (n = 50)	Prodromal AD (n = 53)	All sample
YKL-40/sTREM2, r	0.524**	0.36	0.569^t^	0.438*	0.53**	0.406***
YKL-40/PGRN, r	−0.332	0.420*	0.294	0.042	0.384*	0.171
sTREM2/PGRN, r	0.162	0.459	0.463	0.276	0.499**	0.337**
YKL-40/Aβ_1–42_, r	0.423*	−0.073	−0.331	0.031	0.101	−0.163*
YKL-40/t-tau, r	0.263	0.600***	0.71**	0.317	0.65***	0.572***
YKL-40/p-tau, r	0.285	0.627***	0.778***	0.306	0.591***	0.589***
sTREM2/Aβ_1–42_, r	0.231	0.196	0.042	0.389^t^	0.409	0.152
sTREM2/t-tau, r	0.406*	0.235	0.459	0.078	0.418*	0.179*
sTREM2/p-tau, r	0.531**	0.272	0.455	0.155	0.368^t^	0.204**
PGRN/Aβ_1–42_, r	−0.378^t^	−0.009	−0.129	−0.074	0.210	−0.108
PGRN/t-tau r	0.126	0.339	0.263	0.240	0.393*	0.256***
PGRN/p-tau, r	0.075	0.415^t^	0.449	0.236	0.394	0.294***

r values in bold represent the significant correlations between biomarkers. Partial correlations were adjusted by age for all biomarkers and sex in the case of sTREM2. P-values were adjusted by Bonferroni correction for multiple comparisons (9 comparisons for this analysis).

*p < 0.05.

**p < 0.01.

***p < 0.001.

^t^p-value between 0.09 and 0.05.

### Relationship between glial and core AD biomarkers

Table 2 and Supplementary Fig. S2 show the different correlations between glial and core AD biomarkers in CSF. YKL-40 correlated with t-tau and p-tau in prodDLB (r = 0.71 and r = 0.778, both p < 0.001), and DLB (r = 0.6 and r = 0.627, both p < 0.001). sTREM2 and PGRN levels did not correlate with Aβ_1–42_, t-tau or p-tau in the DLB groups.

### Influence of AD copathology on CSF glial markers in DLB

**Figure 3:**
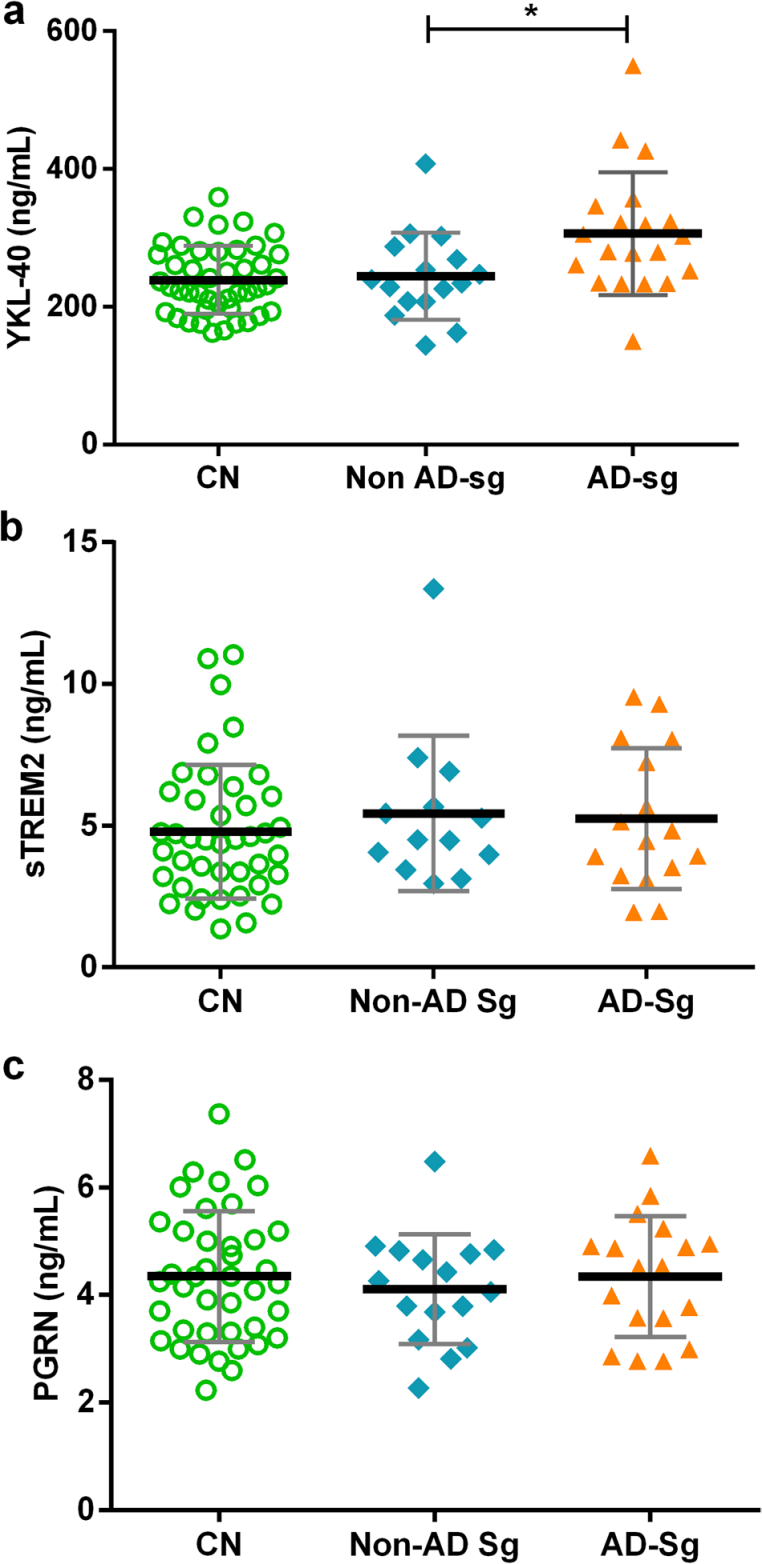
Influence of AD copathology on CSF glial markers in DLB patients. *p-value < 0.05. **p-value = 0.009. Thicker horizontal bars represent the mean while whiskers represent the SD. CN: cognitively normal controls, DLB: Dementia with Lewy Bodies, Non AD-Sg: DLB patients with core CSF biomarkers non-suggestive of concomitant AD copathology (t-tau/Aβ_1–42_ ratio < 0.52). AD-Sg: DLB patients with core CSF biomarkers suggestive of concomitant AD copathology (t-tau/Aβ_1–42_ ratio > 0.52). (**a**–**c**) CSF YKL-40, sTREM2 and PGRN levels in CN and DLB patients with and without AD copathology according to the t-tau/Aβ_1–42_ ratio.

We next analyzed levels of YKL-40, sTREM2 and PGRN in DLB patients according to the presence or absence of a CSF AD profile based on the ratio t-tau/Aβ_1–42_^30^. We found that levels of YKL-40 in DLB patients with a t-tau/Aβ_1–42_ ratio indicative of AD pathophysiology (>0.52) were higher than those with a normal t-tau/Aβ_1–42_ ratio (<0.52, Fig. 3a, p = 0.04). There were no differences in the CSF levels of sTREM2 or PGRN in DLB patients when stratifying by the tau/Aβ_1–42_ ratio (Fig. 3b,c). Suppl. Table 1 shows demographic and clinical data from those patients. We found similar results when analyzing together DLB and prodDLB groups (data nor shown).

**Figure 4:**
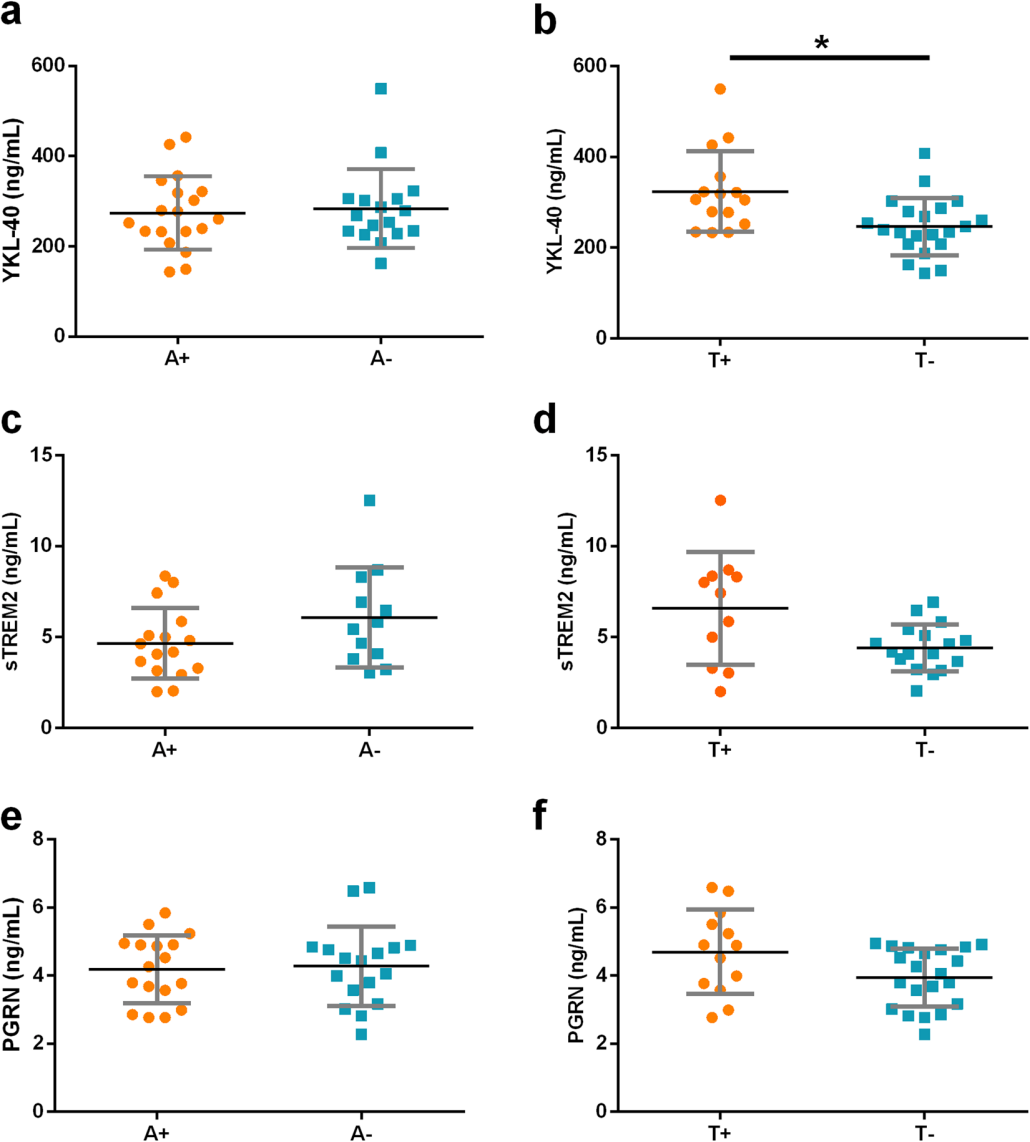
CSF glial markers in DLB patients according to A/T classification. *p-value < 0.05. Thicker horizontal bars represent the mean while whiskers represent the SD. DLB patients were stratified by levels of Aβ_1–42_ and t-tau in CSF: A+: decreased CSF levels of Aβ_1–42_, A−: normal CSF levels of Aβ_1–42_, T+ increased CSF levels of p-tau, T− normal CSF levels of p-tau.

To further investigate the influence of amyloid and tau pathology on the CSF levels of glial markers in DLB we stratified patients according to the A/T scheme^31,32^. We did not find differences in glial markers between A+ vs. A− DLB patients. T+ DLB patients had higher levels of YKL40 than T- DLB patients (p = 0.007, Fig. 4b). T+ DLB patients showed also a trend for higher levels of sTREM2 and PGRN (p = 0.07 and p = 0.09 respectively, Fig. 4d,f).

## Discussion

The main finding of this study is that YKL-40 levels are elevated in CSF in DLB patients only when there is a CSF profile indicative of concomitant AD pathology. The glial markers YKL-40, sTREM2 and PGRN are not increased in CSF in DLB when comparing with CN. We also measured for the first time these glial markers in the prodromal phase of DLB and found no increase at this stage of the disease compared to CN and later stages of DLB.

The lack of increase in CSF YKL-40 in the whole DLB group agrees with previous findings in PD and DLB^33–37^, supporting the absence of an increase of this protein in CSF in synucleinopathies. This may suggest a lack of astroglial activation following the α-synuclein pathology. On the other hand, our group and others have shown an increase of CSF YKL-40 in AD and also FTLD-related syndromes early in the disease course^5,7,8,10,35,38,39^. This contrast between synuclein- and tau-related neurodegenerative dementias suggests that YKL-40 is more involved with this second group of disorders. Moreover, we demonstrate an increase in YKL-40 in DLB patients with concomitant AD copathology when compared with DLB patients without AD copathology, suggesting that it is comorbid AD what is driving astrocytic activation in DLB. In agreement with this finding, we found that YKL-40 levels were highly correlated with t-tau and p-tau levels in DLB groups and that YKL-40 was increased in T+ DLB patients. One possible explanation to the difference in CSF YKL-40 between AD and DLB is that the α-synuclein inclusions observed in astrocytes in DLB may influence the astrocytic response toward neurodegeneration compared to tauopathies such as AD and FTLD^40,41^. In addition, the astrocytic response against pathologic protein deposition in DLB seems to be linked to the presence of pathologic tau (p-tau) in the presence of concomitant AD pathology.

This is the first study that reports the CSF sTREM2 levels in DLB patients. We found higher levels of this protein in DLB compared to AD, but this difference did not survive correction for multiple testing, perhaps due to the relatively small sample size that limited the statistical power. Although TREM2 has not been previously investigated in postmortem DLB, some studies have shown higher levels of TREM2 in brains of PD patients and in PD murine models^42–44^. This could support the hypothesis that TREM2 is elevated in synucleinopathies in contrast to YKL-40. Nevertheless, we could not find differences in sTREM2 levels between DLB and CN. We did not find differences either when stratifying the DLB group by CSF AD profile neither a correlation between sTREM2 and t-tau and p-tau in DLB groups, indicating that the levels of sTREM2 in DLB are independent of neurodegeneration due to AD. We could not replicate previous studies showing higher levels of sTREM2 in AD^14,17^, with only a trend toward higher levels in prodromal AD. These results are possible due to the small number of patients included.

Finally, as previously reported in a smaller study^33^ we did not find any difference in the levels of PGRN protein in CSF in DLB or an influence of AD copathology.

One of the strengths of this study is that we included a group of patients with prodromal DLB. Although there are no established criteria for this stage of the disease, we included only those patients that converted to DLB during the follow-up. YKL-40, sTREM2 and PGRN levels have never been investigated in CSF in prodromal DLB, nevertheless, we did not detect any increase in this stage, indicating that these glial markers do not change significantly early in the disease course. Nonetheless, it is of value to include prodromal DLB patients in biomarker studies, not only to find markers of early disease stage, but also to generate new hypothesis regarding the pathophysiology of the disease. This study has some limitations: the study is based on CSF biomarkers in a single-center cohort and needs validation in a larger independent cohort, the sample size is relatively small, relied on clinical diagnosis and neuropathological confirmation was not available.

In summary, we report that DLB and AD show different patterns of glial activation markers in CSF. YKL-40 is only increased in DLB when there is underlying AD pathology and, in contrast to AD, YKL-40 levels are not elevated in prodromal stages. We could not find differences between DLB and healthy subjects in CSF sTREM2 or PGRN levels, although a trend for higher sTREM2 levels was found compared to AD and independently of AD biomarkers. Together, these results suggest a different pattern of glial activation between DLB and AD, which needs further functional and molecular studies to elucidate the differential role of this innate immune response in DLB and its impact on the disease pathogenesis and progression.

## Methods

### Study participants and clinical classification

We prospectively included 207 subjects evaluated at the Memory Unit at Hospital de Sant Pau between January 2009 and October 2017. Patients had the following diagnoses: DLB (n = 37), prodromal DLB (prodDLB, n = 23), AD dementia (AD, n = 50) and prodromal AD (prodAD, n = 53). We also included 44 cognitively normal controls (CN) selected from the Sant Pau Initiative on Neurodegeneration (SPIN) cohort (“https://santpaumemoryunit.com/our-research/spin-cohort/”). To minimize the effect of gender and age, AD and prodAD cases were age- and gender-matched with the DLB and prodDLB cases. All participants received a clinical and formal neuropsychological assessment^45^ and underwent lumbar puncture to obtain CSF as reported elsewhere^30^. DLB patients were evaluated using a previously reported clinical protocol^45–47^. Briefly, the protocol included Minimental State Examination (MMSE), Global Deterioration Scale (GDS), Unified Parkinson Disease Rating Scale – part III (UPDRS-III), Boston Naming Test (BNT), Free and Cued Selective Reminding Test (FCSRT), Visual Object and Space Perception (VOSP), Trail Making Test part A and B (TMT A and B), Neuropsychiatric Inventory (NPI), semantic and phonetic fluencies, Clinician Assessment of Fluctuations (CAF) and One Day Fluctuation Assessment Scale (ODFAS). The neurological evaluation also included a structured questionnaire to interrogate about the features and onset of psychotic symptoms and sleep^47^.

Patients with DLB met consensus criteria for probable DLB^48^. Patients with prodDLB met general criteria for MCI^49^ with at least one sign of α-sinucleinopathy (visual hallucinations, parkinsonism, or REM sleep behaviour disorder (RBD))^50–52^ and had to meet criteria of probable DLB^48^ during the follow up. According to current clinical diagnostic criteria^48^ a diagnosis of DLB excludes a diagnosis of PD by using a one-year rule in which dementia have to be present before or at least during the first year of onset of the parkinsonism. DLB patients with suspected AD copathology were defined according to the ratio t-tau/Aβ_1–42_ (≥0.52; indicative of underlying AD pathology)^30^. We also stratified DLB patients according the A/T scheme^31,32^, considering A+ when CSF levels of Aβ_1–42_ were lower than 550 pg/mL and T+ when CSF levels of p-tau were higher than 61 pg/mL^7^ Patients with AD dementia and prodAD met the NIA-AA criteria^53,54^ and all had a CSF AD profile defined by low Aβ_1–42_ and high t-tau or p-tau levels according to our published cut-offs^7^. CN were volunteers with a normal neuropsychological evaluation for age and education, normal levels of core AD biomarkers in CSF, and no cognitive complaints.

### CSF collection and analyses

CSF was obtained by lumbar puncture as described^7,30^. CSF is collected and processed in polypropylene tubes following international recommendations. The first 2 ml of CSF are transferred to the general laboratory for cell count, and analysis of glucose and protein levels. A further 10 ml are transferred to our laboratory where samples are processed (centrifuged 2000 g at 4 C, during 10 min) and aliquoted within the first two hours after the lumbar puncture. Aliquots are stored at −80 °C until analysis. Levels of core AD biomarkers (Aβ_1–42_, total tau, and phosphorylated tau), YKL-40 and PGRN in CSF were measured using commercially available kits from Fujirebio-Europe (InnotestTM, catalog numbers Ref 81583 (Aβ_1–42_), Ref 81579 (total tau) and Ref 81581 (phosphorylated tau)), Quidel (catalog number Ref 8020) and Adipogen, Inc. (Catalog number AG-45A-0018YEK-KI01), respectively, as previously described^7,33,30^. sTREM2 levels were measured by ELISA using previously described methods^14,17,55^. Samples were diluted 1:5 to measure sTREM2 and PGRN, while were undiluted for the rest of analytes. All samples were randomized across plates, measured in duplicate, and all included samples had an intra-assay coefficient of variation (CV) <15%. Inter-assay CV% was <20% for all the measured proteins (CVs for each assay are shown in suppl. Table S2). The operator was blinded to clinical diagnosis as in previous studies^14,17^.

### *APOE* genotyping

DNA was extracted using standard procedures and *APOE* was genotyped accordingly to previously described methods^56^.

### Statistical analysis

Differences in categorical variables were assessed by Pearson chi-square tests. Normality of the variables was tested by Shapiro-Wilk test. Non-normally distributed variables (sTREM2, YKL-40, t-tau, and p-tau) were log10-transformed to achieve a normal distribution and all the analyses were performed with the log-transformed values. Aβ_1–42_ did not follow a normal distribution even after log-transformation and non-parametric tests were used. Group comparisons between normally distributed values were performed by an analysis of covariance (ANCOVA) adjusting by the effect of age. CSF sTREM2 comparisons were additionally adjusted by the effect of gender. Partial Pearson Product-Moment correlations controlled by age (and gender in CSF sTREM2) were used to test the association between continuous variables. Aβ_1–42_ differences between groups were tested by Kruskal-Wallis and Mann-Whitney tests. Non-parametric correlations (Spearman) were used with variables that did not follow normal distribution (MMSE). Bonferroni *posthoc* correction was applied to adjust for multiple comparisons. We considered 10 comparisons when comparing all the clinical groups together and 9 in the correlations between glial and AD core biomarkers. The level of significance was set at 5% (α = 0.05). All statistical analyses were performed using SPSS software version 21.0 for Windows.

### Ethical approval and consent to participate

All subjects signed the informed consent form to participate in the study and all study protocols were approved by the local ethics committee at Hospital Sant Pau.in accordance to Declaration of Helsinki.

## Supplementary information

Supplementary Material

## Data Availability

The datasets used and/or analysed during the current study are available from the corresponding author on reasonable request.
