# Supplementary material for: Different pattern of CSF glial markers between dementia with Lewy bodies and Alzheimer’s disease
Source: Sci Rep. 2019 May 24;9:7803. doi: 10.1038/s41598-019-44173-8 (PMC6534578; doi:10.1038/s41598-019-44173-8)
Supplement: Supplementary file 1 — Supplementary Material [file 41598_2019_44173_MOESM1_ESM.pdf]

Supplementary Material

Tittle: **Different pattern of CSF glial markers between dementia with Lewy bodies and Alzheimer's disease**

Authors: Estrella Morenas-Rodríguez, M.D.[1,2,3], Daniel Alcolea M.D., Ph.D. [1,2,3], Marc Suárez-Calvet M.D, Ph.D.[4,5], Laia Muñoz-Llahuna BSc[1,2,3], Eduard Vilaplana Ph.D. [1,2,3], Isabel Sala Matavera Ph.D.[1,2,3], Andrea Subirana MSc[1,2,3], Marta Querol-Vilaseca MSc. [1,2,3], María Carmona-Iragui M.D., Ph.D. [1,2,3], Ignacio Illán-Gala M.D. [1,2,3], Roser Ribosa-Nogué M.D. [1,2,3], Rafael Blesa M.D., Ph.D. [1,2,3], Christian Haass, PhD[4,5,6], Juan Fortea M.D., Ph.D. [1,2,3], Alberto Lleó, M.D., Ph.D* [1,2,3].

Affiliations

[1] Memory Unit, Neurology Department, Hospital de la Santa Creu i Sant Pau, Barcelona, [2] Institut d'Investigacions Biomediques Sant Pau – Universitat Autònoma de Barcelona, [3] Centro de Investigación Biomédica en Red en Enfermedades Neurodegenerativas, CIBERNED, Instituto de Salud Carlos III. [4] Chair of Metabolic Biochemistry, Biomedical Center (BMC), Faculty of Medicine, Ludwig-Maximilians-Universität München, Munich, Germany. [5] German Center for Neurodegenerative Diseases (DZNE) Munich, Munich, Germany. [6] Munich Cluster for Systems Neurology (SyNergy), Munich, Germany.

Suppl. table S1. Demographic and basic clinical data from DLB patients

	DLB with AD copathology (n = 20)	DLB without AD copathology (n = 17)	p-value
**Age, y ± SD**	77.1 ± 1.1	75.7 ± 1.2	0.420
**Sex, Females % (n)**	55% (11)	52.9% (9)	1
**MMSE, mean ± SD**	21.9 ± 1.2	24.2 ± 0.8	0.165
**Mean follow-up time (y) ± SD**	2.2 ± 0.5	2.6 ± 0.6	0.568
***APOEε4*, %* (n)**	30% (6)	17.6% (3)	0.462

Supplementary figure S1. Relationship between glial biomarkers by diagnosis

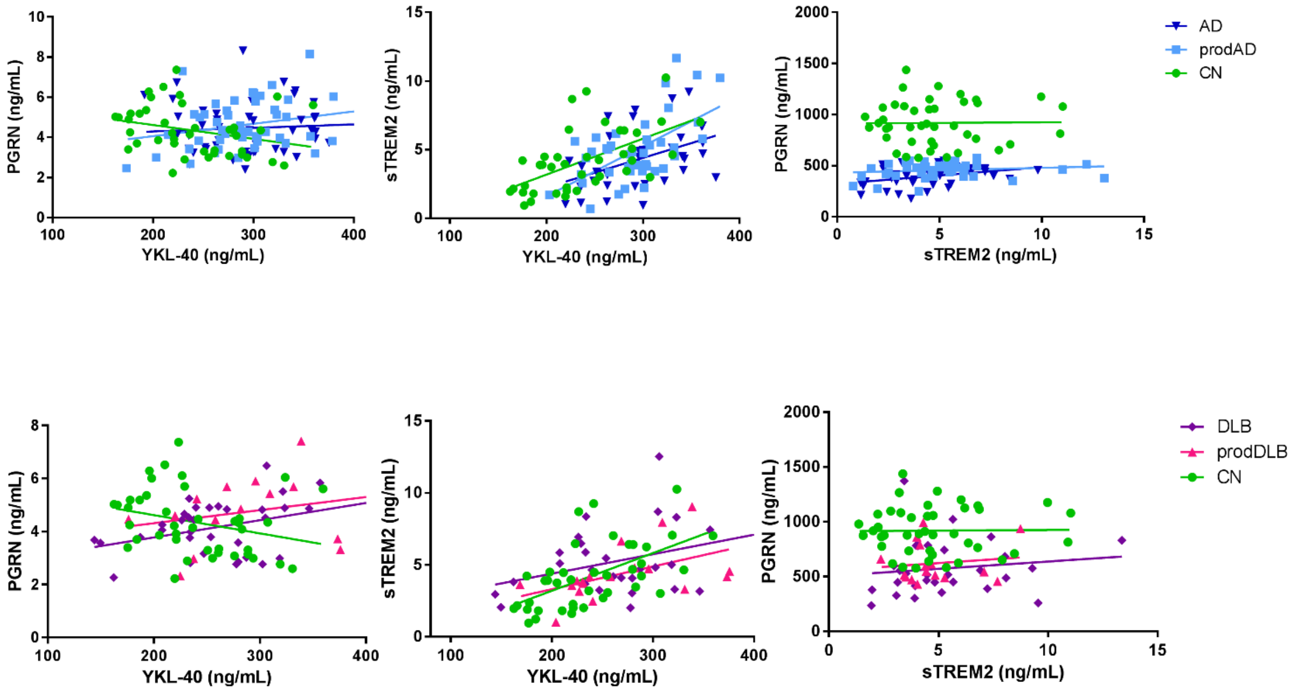

CN: cognitively normal controls, DLB: Dementia with Lewy Bodies, prodDLB: prodromal DLB, AD: Alzheimer's disease, prodAD: prodromal AD.

Supplementary figure S2. Relationship between glial and core AD biomarkers

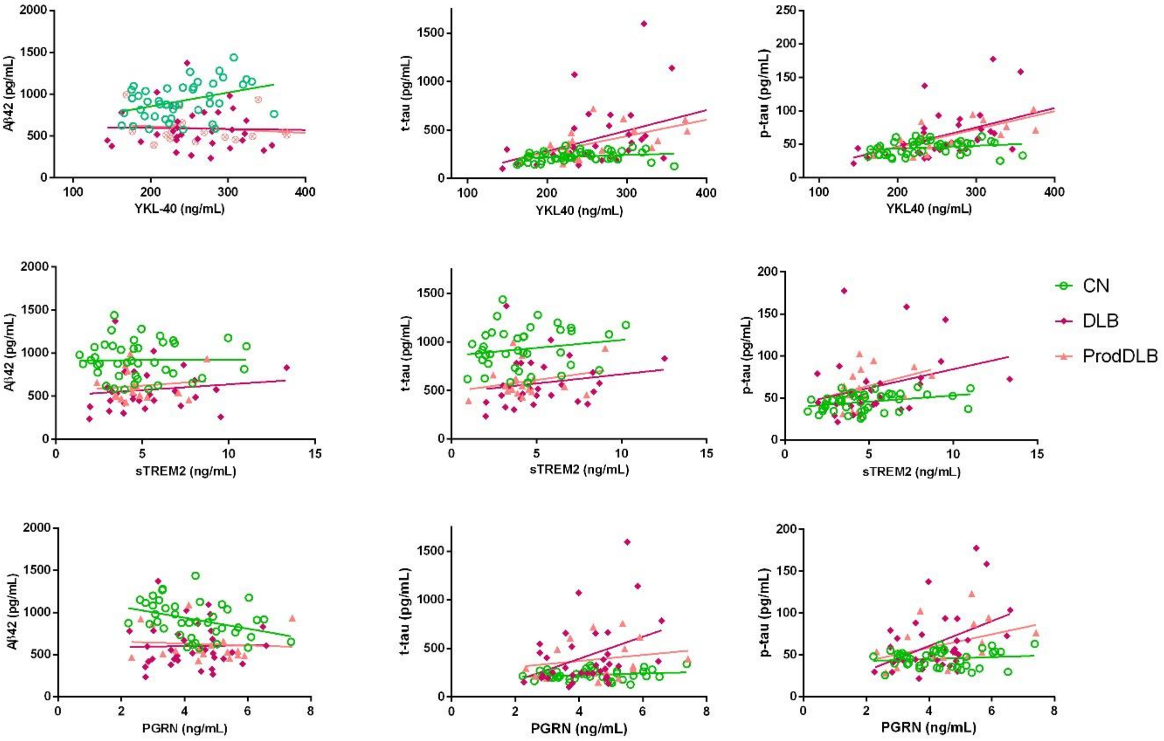

CN: cognitively normal controls, DLB: Dementia with Lewy Bodies, prodDLB: prodromal DLB,

Supplementary methods

Supplementary table S2. Intra and inter-assay CV% per measured protein.

Assay	intra-assay CV%*	inter-assay CV%**
**Aβ1-42**	2.01%	11.07%
**t-tau**	2.01%	10.89%
**p-tau**	1.77%	17.24%
**sTREM2**	3.9%	13.02%
**YKL-40**	4.03%	6.3%
**PGRN**	4.47%	10.7%

* Intra-assay CV% was calculated as the mean of all CV% from the samples included per assay

** We included the following internal controls per assay to calculate the inter-assay CV%. Internal controls are samples with an already known concentration that we included in all the assays. In Braquets there is the mean from all assays and the interplate CV % of each internal control. Aβ1-42: L10-001 (mean: 392.5 pg/mL, CV%: 10.85%), Aβ384 (mean: 359 pg/mL, CV%: 12.48%), Aβ500 (mean: 472,73 pg/mL, CV%: 9.87%). t-tau: tau300 (mean: 297.78 pg/mL, CV%: 10.89%). p-tau: pTau125 (mean: 132.98 pg/mL, CV%: 17.24%). sTREM2: Interplate D (mean: 3.08 ng/mL, CV%: 11.54%) and Interplate

E (mean: 4.44 ng/mL, CV%: 14.51%). YKL-40: YKL40H (mean: 142.84 ng/mL, CV%: 6.3%). PGRN: L10-84 (mean: 3.67 ng/mL, CV%: 15.45%) and L15-033 (mean: 5.12 ng/mL, CV%: 5.12%)

Standard curve calculation per assay

- Standard curves for Innotest assays (Aβ_{1-42}, t-tau and p-tau) were calculated with a 4-parameter logistic regression (provided by the manufacturer).
- Standard curves for sTREM2 assays were calculated by with a 4-parameter logistic regression in the MSD software.
- Standard curves for YKL-40 assays were calculated by a polinomic regression in Excel as recommended by the manufacturer.
- Standard curves for PGRN assays were calculated by a polinomic regression in Excel as recommended by the manufacturer.

All the assays had standard curves with a R squared over 0.95
